# Impact of the POPulation Medicine Multimorbidity Intervention in Xishui County (POPMIX) on suspected asthma patients: protocol for the POPMIX-Asthma cluster-randomized controlled trial

**DOI:** 10.1186/s13063-026-09685-5

**Published:** 2026-04-11

**Authors:** Ke Huang, Xunliang Tong, Xingyao Tang, Qiande Lai, Yuhao Liu, Shiyu Zhang, Zhoutao Zheng, Wenjin Chen, Zhong Cao, Lei Tang, Jinghan Zhao, Liu He, Lirui Jiao, Yingping Wang, Tianying Zhao, Yingchi Luo, Xiangqin Lyu, Qiushi Chen, Sebastian Vollmer, Pascal Geldsetzer, Dean Jamison, Till Bärnighausen, Simiao Chen, Chen Wang, Ting Yang, POPMIX study investigators, Xingyao Tang, Chun Zhang, Yuzhu Ye, Mingqiang Hou, Shengbo Liao, Yuan Zhou, Dong Liu, Chentao Zhong, Yushuang Zhao, Hongxia Mu, Min Liu, Qiong Lyu, Zhengyu Zhang, Weiwei Li, Jian Wang, Tiansheng Lan, Tingyu Liu, Xin Zhou, Bie Yu, Xianping Wang, Yansong Luo, Wenwu Jiang, Wen Zhang, Dongmei Wu, Qingping Luo, Dajun Rao, Cunkun Yang, Feng Zhao, Qin Wang, Linlin Zhang, Can Li, Min Chen, Yufeng Huang, Guanghai Jian, Li Wang, Yunjiang Zheng, Jianghong Linghu, Li Zhong, Xiaoling Ma, Bin Gui, Yan Cheng, Yu Yang, Jing Yan, Xixi Liao, Mingguo Xu, Zhijun Zhao, Qian Han, Panyan Wu, Dandan Tang, Qingqing Wang, Li Feng, Lilan Wang, Lei Si, Peng Zeng, Jianxiu Tian, Weili Fan, Xin Luo, Sumei Feng, Rong Ding, Huadan Yi, Fengxue Lyu, Ludan Yuan, Yuan Liu, Xueshuang Wang, Aihong Duan, Yang Zhao, Yuping Luo, Yicheng Li, Anchun Liang, Chengmei Li, Hu Tang, Anfeng Liu, Xu Wang, Yi Zhong, Wen Zheng, Benwei Zhao, Qianmei Zhang, Xiaojing Tu, Huanhuan Lu, Xiumei Zhao, Yan Chen, Xuemei Yuan, Yanhong Mu, Yao Zhao, Juan Zhang, Qinhui Li, Fuhui Wang, Xiaomin Mu, Yan Hu, Liuyang Chen, Gang Lei, Zhufei Hu, Yan Luo, Shunxia Zhou, Ting Pan, Yuanyin Zhou, Xiaoqin Ren, Yaling Zhou, Jing Zeng, Keping Hu, Xiaoyu Liu, Deguo Yang, Qiong Wan, Cai Zhang, Heng He, Chengping Wang, Yuanzhong Wang, Shuyin Wang, Xuhong Lei, Yushen Yuan, Tailiang Luo, Dazhou Zhu, Anrong Luo, Xianmei Hu, Ya Zhang, Ling Yuan, Senlin Luo, Weili Ma, Yonghua Cao, Yunpeng Cao, Taojin Chen, Wen Chen, Zhiqun Chen, Zhongyi Chen, Huagang Cheng, Xianpan Feng, Taimei Gao, Lizhu Jian, Renxian Jiang, Shengmei Kang, Hang Lei, Shanshan Liu, Ruxun Lu, Xiaojiao Luo, Dongshun Lyu, Guanghong Meng, Jianping Mu, Xuerou Mu, Hongxia Qian, Huimin Ren, Juan Wang, Li Wang, Loutao Wang, Qin Wang, Wei Wang, Ya Wang, Yan Wang, Dongjie Wu, Xiaoxia Xu, Jing Yang, Qian Yu, Dan Yuan, Yanlu Yuan, Yu Yuan, Hongying Yue, Yuanmin Yue, Li Zhang, Zhizhong Zhang, Jiaqin Zhao, Yuanxin Zhao, Yue Zhong, Lejie Zhou, Lufang Yang, Xue Chen, Lihui Ren, Guixiang Gong, Ping Shi, Jiangshun Tan, Zhonghang Yu, Lijun Mu, Meilin Chen, Su Yu, Lin Li, Xianbi Mu, Jianxia Wu, Mei Zhao, Jiangmei Chen, Jing Huang, Yanfang Li, Huayan Yang, Jiaheng Yang, Xingqi Yuan, Yunhao Zhou, Mengting Ye, Junlin Zhang, Taijun Luo, Shuai Wang, Ju Hu, Gang Yuan, Shili Mu, Zhongxiang Yuan, Huili He, Jiali Li, Chunlan Liu, Kailiang Cheng, Quan Liu, Huade Yang, Shaojun Zhang, Bo Chen, Guishan Ying, Xumei Zhao, Zhi He, Gan Feng, Huanhuan Guo, Hongling Yuan, Fuyou Yuan, Wei Wang, Lang Chen, Liping Zhao, Jiajun Ning, Jie Feng, Yuxian Chen, Jin Chen, Jing Qi, Yuanting Li, Lingli Zhao, Guo Wang, Ziyi Wang, Huaishu Zhong, Jie Wang, Gang Chen, Xueqin Mu, Yuanfeng Gong, Xinan Lu, Zhengli Chen, Tianwei Chen, Xiaoping Chen, Runqin Mu, Xiaoyi Deng, Yu Duan, Xiujuan Dong, Wangyong Yan, Jiayong Rao, Mingfang Wang, Xiangcai Chen, Zhongquan Wang, Lanfang Zhang, Jianghua Luo, Kaijie Huang, Ye Chen, Jin’e Li, Li Huang, Ju Chen, Hao Huang, Qingqun Long, Yan Chen, Ling Jiang, Man Ding, Xiaolin Yu, Tongjie Wei, Zhengqin Liu, Kaifu Si, Yuxian Mu, Yan Huang, Qian Yang, Tao Xu, Xiaorong Yuan, Cailun Zhao, Minli Chen, Rongfang Luo, Ling Chen, Ying Zhang, Guiwei Yuan, Qianyi Shui, Yong Wu, Qian Huang, Changli Wu, Nan Zhang, Chaojiang Wu, Defei Chen, Wu Xu, Linhui He, Wanlun Zhang, Jinsong Chen, Jin Yuan, Rujun Li, Minghe Wang, Chunmei Zhang, Yueshan Wang, Lang Liao, Anqian Feng, Dengyang Wang, Zelin Deng, Hupiao Yang, Kaimeng You, Lijuan Deng, Qiong Huang, Xiaoqing Zhang, Zhengqian Zhu, Xiaoyan Liao, Yongbo Song, Yushui Yuan, Qinghong Cai, Lu Liu, Hong Yu, Xue Luo, Xiaoshan Huang, Qin Hu, Dian Yuan, Zengye Zhao, Jiemin Zeng, Zhaoyan Huang, Peng Luo, Taibo Luo, Wenwu Wu, Guangni Liu, Gengning Zhang, Mingyuan Ren, Mei Yang, Zhongjin Wang, Rongping Yuan, Yunfeng Yuan, Anquan Yuan, Yunqiang Chen, Hongxian Liu, Xingyu He, Tongxiang Zhang, Mingkai Xu, Minqing Hu, Chunyi Qian, Yunfen Zhang, Yueguang Lei, Jun Wang, Xiaoju Yuan, Changxian Yang, Ai Xiang, Minjing Zhao, Xiaomei Ren, Guimei Li, Huiying Ren, Ju Teng, Yu Chen, Jing Yuan, Shunfeng Mu, Xiangyu Zhang, Ye Li, Yuwei Sun, Shangjing Wang, Fangping He, Guoxiang Wang, Wei Cai, Weijiang Tian, Tingfen Cai, Zhao Chen, Dakai Mu, Yufu Mu, Cai Cheng, Xiaofang Pan, Fang Wang, Mingqian Zhao, Qijun Liu, Bangqi Yang, Ye Yu, Ting Min, Xiaoya Wang, Jun Li, Yang Zhang, Yan Wang, Lihong Zhao, Zhihua Luo, Hao Yuan, Huihua Ruan, Jian Wu, Chengfen Wang, Dezhi Zhang, Yunfang Zhang, Weining Ao, Pan Zhao, Yunxia Liang, Yicheng Zhao, Xiaosong Mu, Lyufang Luo, Ziyi Zhang, Wei Huang, Jun Cao, Huan Li, Jun Liu, Liye Yuan, Na Zhao, Ju Wang, Qiuyan Duan, Weizhong Chen, Lirong Zhao, Jiangbo Wu, Liqin Deng, Kunyu Wang, Zhengguo Yan, Chaolian Wang, Can Liu, Quan Cao, Gang Ma, Youyong Lu, Yuan Zhang, Kaijing Xiang, Yixiang Wang, Xingye Zhu, Jiamei Wang, Demin Hu, Dongmin Li, Peng Chen, Chao Tian, Lu Wang, Mei Wang, Lulu Xiao, Lingling Yu, Min Wei, Chengcheng Yang, Limei Zhang, Rong Zhao, Chongguang Zhong, Yan Xiao, Jian Luo, Hongfa Chen, Pengju Dai, Jin Liu, Yingtao Luo, Guangjie Ma, Shiyou Mu, Zhengli Rao, Deqin Wen, Guowang Wu, Yong Hu, Xiaorong Shi, Shaofen Huang, Ya Chen, Wenmao Zhang, Min Chen, Xin Luo, Hongqiong Ruan, Liming Liu, Lihui Fan, Xiaoyan Zou, Huizhi He, Xinggao Yang, Linfei Wang, Hong Wang, Wenxiang Li, Jialian Wu, Jinyong Yang, Jianying Wu, Guangliang Liu, Xiaolin Xiong, Gang Yang, Ping Feng, Cheng Luo, Qiwei Zhang, Qihui Yang, Taoshan Ni, Rensong Tian, Li Zhang, Yuanmei Zhang, Hui Bi, Bijue Huang, Yu Yan, Shan Chen, Xinghui Chen, Jiangfei Wang, Guanglun Zou, Junting Liu, Rongjiang Zhao, Li Yuan, Ruyin Yuan, Jianbing Li, Aiping Wang, Daijie Wang, Mei Yang, Kaiyan Si, Peng Wang, Yan Linghu, Tuye Yuan, Zhiyuan Yuan, Weiwei Qian, Daimei Wang, Rongqin Ren, Dong Dai, Qianhua Deng, Yan Xu, Jiali Zhang, Qingyan Bai, Yingwen Zhang, Xingke Wang, Qiang Liu, Liyuan Feng, Fancha Wang, Shiwen Wang, Xiangguang Liu, Runping Liu, Xiaoying Wang, Daizhi Wang, Huihai Cao, Jiang Yuan, Qinhui Cao, Min Luo, Hao Hou, Lu Kong, Zhu Ren, Yu Zhou, Xiaoyan Yuan, Hong Liu, Shixian Gong, Yonghong Zhao, Rusheng Wang, Ji Chen, Fuping Bai, Yang Zhou, Wenbi Chen, Yu Sun, Qunying Xu, Gang Zheng, Yong Wang, Juan Chen, Ling Zhong, Ji Ren, Qiling Zhu, Zheng Yang, Yongsong Chen, Zhonghui Huang, Xiaojiang Zhang, Junyan Yu, Panting Yu, Qun Wang, Qimei Yu, Hao Zhou, Yuanmei Lu, Yiyu Wang, Min Huang, Feng Wu, Jiangyu Yuan, Guangjun Xiong, Jian Zhang, Hong Zhou, Fenfang Liu, Yuqin Chen, Xiaoli Ye, Qingli Luo, Qijie Dai, Yunxian Feng, Tianmei Huang, Yunfeng Xia, Lei Zhao, Anshui Wang, Fuxiu Yang, Minqin Hu, Tingting Yuan, Song Zhou, Lin Yuan, Mingjian Yuan, Maozhao Wen, Hongming Zhao, Hongbo Zhao, Yuanchao Zhao, Binyuan Yuan, Hua Xiong, Qiyong Yuan, Qin Yuan, Xingqin Zhao, Qifu Luo, Qigui Luo, Tulin Yuan, Xianglin Huang, Chun Luo, Dehong Wang, Rongwei Zhang, Xingji Chen, Zhong’en Wang, Deping Yuan, Qihua Luo, Huahua Li, Yueming Wang, Jian Chen, Wei Teng, Jisheng Wang, Mingli He, Shan Wu, Junjun Xia, Tingxian Mao, Tingmin Luo, Sihong Yi, Ruixue Li, Kang Yang, Chengshang Ao, Xu Zhong, Yang Liu, Langsha Chen, Huan Luo, Qingxia Wang, Chen Chen, Wenqiang Wu, Yumei Wu, Limei Feng, Caixian Lei, Kai Li, Youqun Chen, Lulu Zhao, Han Ren, Juhang Yang, Shanshan Luo, Xiaoyi Zhang, Huiqin Yuan, Li Chen, Guishuang Liu, Jiakuan Ren, Dongqiao Jiang, Yi Yuan, Dengmin Ren, Xiaojiang Mu, Xue Yang, Wuyao He, Wanyi Hu, Piaopiao Huang, Guijiang Li, Jinman Li, Suosuo Li, Yiling Lu, Yuqi Lu, Huan Luo, Qingqing Luo, Wenqin Mu, Daolin Qin, Jinlan Quan, Yingying Tang, Junbao Wang, Lingling Wang, Rui Xu, Chenxi Yang, Dengqin Yang, Liqin Yang, Qingting Zhang, Lei Zhao, Lin Zhao, Jingmei Zhou, Lijuan Zuo, Xiaoning Xia, Yujia Xie, Hangyu Chen, Li Yang, Yu Zhao, Sisi Li, Juan Pan, Ju Yang, Minjia Zhao, Meiyuan Zhou, Guimei Hu, Chunqin Huang, Haiyan Long, Zeyuan Luo, Donghua Pan, Xuelang Ying, Yanyan Zhang, Yuting Zhang, Hong Zhao, Yunli Zhou, Bijin Zhu, Kang Chen, Kaidi Ding, Jiacheng Dong, Tai Fu, Qin Li, Zaijing Liu, Siqi Huang, Lingli Ou, Zhuoting Tan, Yan Tang, Qingzhong Wu, Xinyi Xu, Bingyang Xu, Yulin Zhang, Shuangshuang Zhou, Zena Zhu, Jiancao Zuo, Guangxianfeng Chen, Jiahui Luo, Zhiqi Duan, Shangrong Gu, Binyao Ou, Xingtai Zhao, Yanran He, Xingli Liao, Shunxin Shi, Cheng Wang, Jiayu Wang, Rongmei Wang, Ruirui Zhang, Yuhan Zhou, Serena Luo, Jiwei Jin, Xiaoqi Shi, Ziqianqian Yang, Xiaoxue An, Jiwei Jin, Mingli Huang, Yinghua Mu, Xiaozheng Wang, Songlin Zhang, Xue Zhou, Ruirui Ao, Shaohuan Bao, Yating Jiang, Yijun Liu, Zhirui Nie, Dingyuan Rao, Jiangqin Wan, Xue Wang, Die Wei, Lijin Xie, Jianan Xu, Xuerong Zhang, Die Deng, Jingyu Fu, Shunli Guo, Binhan He, Sitong Liu, Lin Liu, Linqu Qin, Teng Tian, Kehao Wang, Qirui Wu, Laitian Ye, Xiao Zhang, Tiantian Zhou, Sitong Zhou, Jiajia Liu, Huali Yin, Yue Luo, Jianxiu Bai, Yuchan Li, Xinglian Zhang, Shenghui Zhu, Xue Chen, Dan Jiang, Xiaoqin Yang, Changnan Zhao, Fangjing Geng, Jia Li, Jie Wang, Wenfan Hu, Xingling Li, Yumei Lu, Zhuying Long, Junxue Qian, Guoxian Shi, Ruoxi Wang, Guangjian Wu, Guangyan Zhang, Rongrong Zhou, Hongyu Hu, Zhaoyi Ren, Lingxuan Shi, Meng Zeng, Shengsheng Li, Ying Qin, Yi Zhou, Tianyu Liu, Jia He, Jinan Li, Mingyuan Mao, Jiayi Wang, Ronghui Ma, Bin Li

**Affiliations:** 1 Department of Pulmonary and Critical Care Medicine, China-Japan Friendship Hospital, No.2, Yinghua Donglu Street, Chaoyang District, Beijing, China; 2 National Center for Respiratory Medicine, Beijing, China; 3 State Key Laboratory of Respiratory Health and Multimorbidity, Beijing, China; 4 Department of Pulmonary and Critical Care Medicine, National Center of Gerontology, Beijing Hospital, Institute of Geriatric Medicine, Chinese Academy of Medical Sciences, Beijing, China; 5 Department of Pulmonary and Critical Care Medicine, People’s Hospital of Xishui County, Zunyi, Guizhou China; 6 School of Population Medicine and Public Health, Chinese Academy of Medical Sciences & Peking Union Medical College, Beijige Santiao 31, Dongcheng District, Beijing, China; 7 School of Health Policy and Management, Chinese Academy of Medical Sciences & Peking Union Medical College, Beijing, China; 8 Heidelberg Institute of Global Health, Faculty of Medicine and University Hospital, Heidelberg University, Heidelberg, Germany; 9 Guizhou Medical University, Guiyang, Guizhou China; 10 Department of Health Policy and Management, Gillings School of Global Public Health, University of North Carolina at Chapel Hill, Chapel Hill, NC USA; 11 Center for Disease Control and Prevention of Xishui County, Zunyi, Guizhou China; 12 The Harold and Inge Marcus Department of Industrial and Manufacturing Engineering, The Pennsylvania State University, University Park, PA USA; 13 Department of Economics and Centre for Modern Indian Studies, University of Goettingen, Göttingen, Germany; 14 Division of Primary Care and Population Health, Department of Medicine, Stanford University, Stanford, CA USA; 15 Department of Epidemiology and Population Health, Stanford University, Stanford, CA USA; 16 Department of Epidemiology and Biostatistics and Institute for Global Health Sciences, University of California, San Francisco, CA USA; 17 Department of Global and Population Health, Harvard T.H. Chan School of Public Health, Harvard University, Boston, USA

**Keywords:** Population medicine, Asthma, Mmultimorbidity, Suspected asthma patients, cRCT

## Abstract

**Background:**

Asthma is a common chronic disease responsible for a considerable disease burden in China and around the world. Despite its burden, there is substantial unmet need for asthma care, including screening, diagnosis, treatment, and management. Symptom-based screening for asthma could support identification of undiagnosed asthma patients, as well as reference to higher-level hospitals for formal diagnoses and treatment. This study focuses on identifying suspected asthma patients and encouraging them to seek formal diagnoses and treatment. This approach aligns with the novel concept of population medicine, which aims to maximize overall population health rather than focusing on individual patients within the health system.

**Methods:**

We are conducting a two-arm population-based stratified clustered randomized controlled trial (cRCT) to evaluate the effectiveness of a population medicine multimorbidity intervention package. The intervention integrates community screening, chronic disease management, patient education, digital follow-up, and team-based care. The trial is being implemented in Xishui County, Guizhou Province, a mountainous low-resource county in Southwestern China, covering 26 townships and more than 300,000 permanent residents. We considered each of the 26 townships in Xishui County as a cluster and stratified them into large and small townships based on population size. Townships with an above-average population were designated as “large,” and those with a below-average population were designated as “small.” We randomized the same number of residents in each township stratum (large and small) to undergo the European Community Respiratory Health Survey (ECRHS) for identifying suspected asthma patients. Individuals identified as suspected asthma patients were considered study participants and subsequently enrolled in the intervention or control arm. All participants in the intervention arm are followed for one year, with one telephone follow-up at month three and in-person follow-ups at months six and 12, while participants in the control arm are followed only at baseline and 12 months. Primary outcomes include the number of chronic conditions controlled, whether the participant received lung function testing, and Asthma Control Test (ACT) score. In addition, we are evaluating 42 secondary outcomes covering physiological and functional indicators such as lung function, health-related quality of life, mental health, behavioral risk factors, healthcare utilization, productivity loss, knowledge of asthma and chronic obstructive pulmonary disease (COPD), and care cascade indicators for asthma and other chronic diseases.

**Discussion:**

This cRCT has been featured as an important case study in the *Lancet* Commission on Investing in Health report to evaluate the effectiveness of the integrated intervention package on priority conditions. The trial was designed under population medicine principles, with an aim providing holistic care and enhancing the overall health status of suspected asthma patients. The results of the trial will inform the next generation of multimorbidity management and population medicine practices among global health authorities and practitioners.

**Trial registration:**

ClinicalTrials.gov Identifier: NCT06457009. Registered on June 7, 2024.

**Supplementary Information:**

The online version contains supplementary material available at 10.1186/s13063-026-09685-5.

## Contributions to the literature

- POPMIX-Asthma trial evaluates a novel, multi-component population medicine intervention for managing asthma and multimorbidity among suspected (not yet diagnosed) patients in a resource-limited rural Chinese county.
- POPMIX-Asthma explores population-based asthma screening in low-resource setting where gold standards of diagnosing asthma are usually not feasible.
- It introduces and tests a “pay-for-population” incentive mechanism, designed to motivate primary and community care providers to proactively engage in community-wide screening, diagnosis, and management.
- POPMIX-Asthma will generate critical evidence on the effectiveness of integrating community screening, digital health tools, and team-based care to improve the care cascade for asthma and its common co-occurring conditions.
- Findings will inform the scalability of population-centered, rather than patient-centered, care models for asthma and related multimorbidity in low-resource settings globally.

## Background and rationale

Medicine is undergoing a shift from focusing solely on individual patients to addressing the health of entire populations, as many people face an unidentified and unaddressed need for healthcare. The concept of population medicine emphasizes the significance of maximizing overall population health and welfare [1]. Studies grounded in population medicine focus on priority conditions and identifying cost-effective interventions and disease management strategies that have the potential to substantially improve population wellbeing. The recent *Lancet *Commission on Investing in Health has identified major drivers of the life expectancy gap between China and the North Atlantic region, the latter of which has the highest life expectancy worldwide. Tobacco-related non-communicable diseases (NCDs) collectively represent one of the major causes of life expectancy gaps in China [2]. This suggests that common chronic respiratory diseases, such as asthma and chronic obstructive pulmonary disease (COPD), should receive greater attention, as health systems often lack strategies for population-level screening, diagnosis, treatment, and management of these conditions [3–5].

Asthma is a common chronic airway disease, responsible for a considerable burden of disease globally [6, 7]. The prevalence of asthma is generally higher in regions with low socio-economic profiles [4, 8, 9]. In China, the overall prevalence of asthma among adults aged 20 years and above was estimated to be 4.2%, or 45.7 million individuals, based on the China Pulmonary Health Survey (CPHS) Survey conducted between 2012 and 2015 [4], and the prevalence of asthma with airflow limitation was 1.1%, representing 13.1 million adults. Asthma with airflow limitation, a progressive form of the condition, may be associated with decreased quality of life and increased probability of future exacerbation, and is more common in rural and low-resource areas [4, 10]. The lack of a therapeutic cure for asthma makes it essential to understand the condition’s risk factors and explore strategies for large-scale asthma screening among the general population. Although asthma’s causal mechanisms are still under research, risk factors for the condition are well-documented and include cigarette smoking, parental smoking, and high body mass index (BMI) [6]. Potential strategies for population-based intervention include primary and community care approaches and chronic disease management. Screening and identification of suspected asthma patients are especially important in low-resource settings given that these areas often suffer from poor medical facilities and misdiagnoses.

Despite a worrying epidemiological pattern of asthma in China, previous studies have shown low healthcare utilization among asthma patients. Among detected patients in the CPHS [4], only 28.8% reported ever being diagnosed by a physician, 23.4% reported receiving a pulmonary function test, and 5.6% had been treated with inhaled corticosteroids. Additionally, a substantial proportion of detected patients in CPHS had reported at least one emergency room visit (15.5%) and at least one hospital admission due to exacerbation of respiratory symptoms (7.2%) within the preceding year. This evidence of poor healthcare utilization points toward a substantial unmet need for asthma-related care, including testing, diagnoses, treatment, and control.

Unfortunately, the complex natural history of asthma and heterogeneity of presentations confound diagnosis attempts at the level of primary care and in low-resource settings, where misdiagnoses are common [5, 6]. Researchers have therefore conducted multiple epidemiological studies using standardized symptom-based screening questionnaires to identify asthma patients [5], including the European Community Respiratory Health Survey (ECRHS) [11–13], the International Study of Asthma and Allergies in Childhood (ISAAC) [14–18], the International Study of Wheezing in Infants (EISL) [19, 20], the World Health Survey (WHS) [21], and CPHS [4]. The most recent nationwide asthma prevalence survey in China was CPHS, which used the ECRHS screening questionnaire for asthma. The ECRHS screening questionnaire has been widely used in other prior studies, including WHS [22] and the Global Burden of Disease Study [9], and was validated in the Chinese context in CPHS [4] and various regional asthma surveys [22–24]. The CPHS adopted the ECRHS screening questionnaire, which includes standardized symptom-based questions designed for large-scale epidemiological studies. This enabled researchers to identify individuals with possible asthma symptoms efficiently and conduct screening at the community level.

The *Lancet* Commission on Investing in Health [2] report has identified cost-effective modular approaches to care for tobacco-related NCDs, which collectively represent the third leading cause of the life expectancy gap between China and the North Atlantic region. Since asthma is a common chronic disease that shares risk factors with COPD, hypertension, and type 2 diabetes mellitus, satisfying the unmet healthcare need among suspected asthma patients and managing these patients’ conditions with an integrated multimorbidity intervention package is likely to make an outsized contribution to population health and wellbeing. The intervention strategies comprising the package, which include community-based screening, health education, and smoking cessation interventions, have been proven cost-effective and feasible in low-resource settings.

### Objectives

This investigation is nested within the Population Medicine Multimorbidity Interventions in Xishui (POPMIX) project—a collaborative effort focused on crafting and validating practical, multi-pronged approaches to managing chronic diseases and co-occurring conditions in communities in a resource-limited rural county in China. Our specific aim is to explore how a bundled intervention strategy impacts three core areas for individuals suspected of having asthma: their engagement with the care process (care cascade), symptom relief, and general health.

Central to our evaluation are three primary metrics: the number of chronic conditions successfully managed, whether lung function testing was conducted, and the score on the Asthma Control Test (ACT). Beyond these primary targets, the trial’s large-scale, community-based design allows us to ambitiously probe a diverse set of secondary outcomes. These include physiological and functional indicators such as lung function, health-related quality of life, mental health, behavioral risk factors, healthcare utilization, productivity loss, knowledge, and care cascade indicators for asthma and other chronic diseases.

### Trial design

**Fig. 1:**
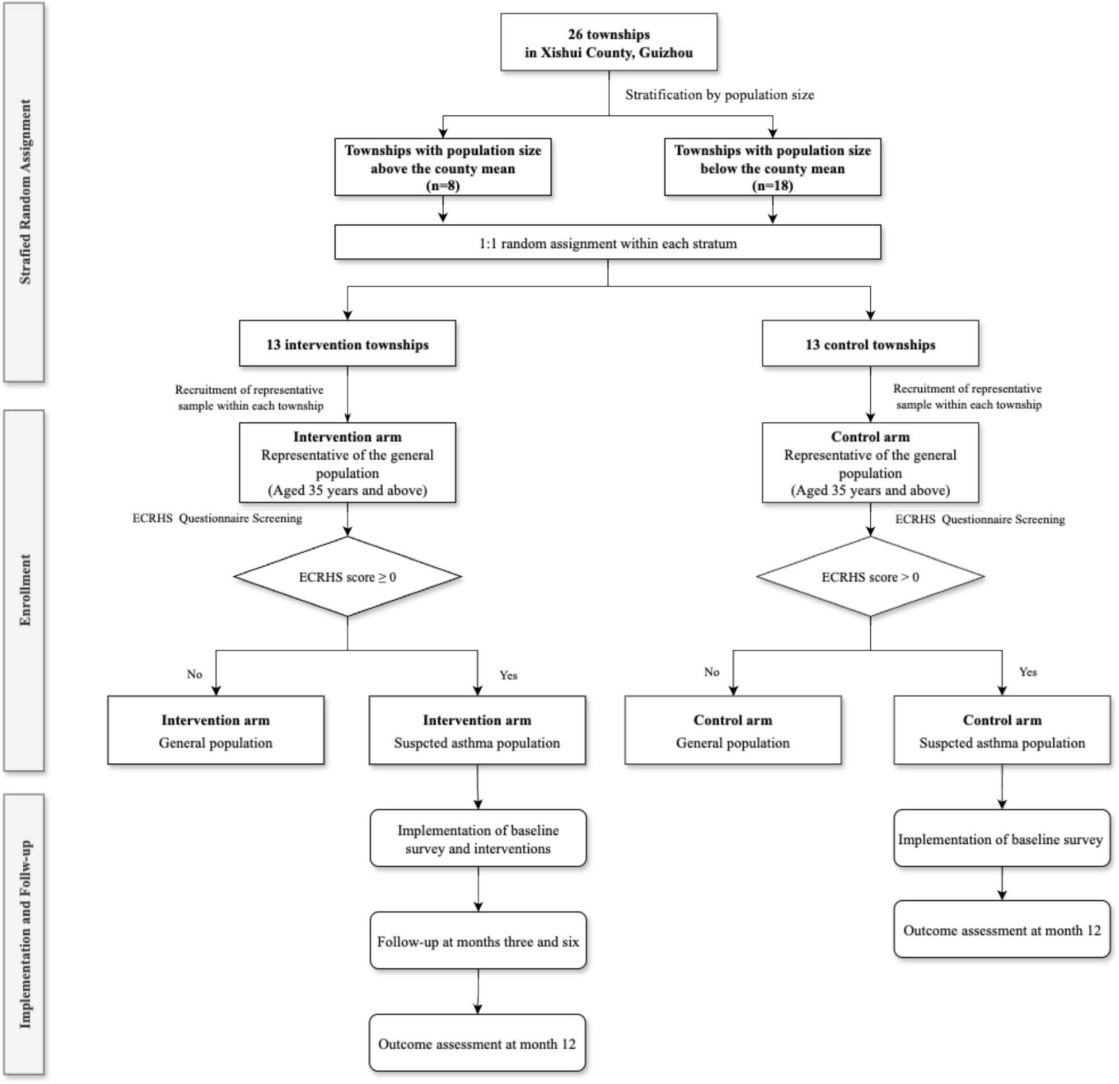
Flowchart of POPMIX-Asthma trial

This research utilized a two-group, parallel-design, stratified cluster-randomized controlled trial (cRCT) framework and was carried out in Xishui, Guizhou, China (Fig. 1). The trial protocol was crafted following the guidelines of the Standard Protocol Items: Recommendations for Interventional Trials (SPIRIT) 2025. A comprehensive outline of the SPIRIT Checklist applicable to this protocol is included in Supplementary Material, Additional File 1. Patient and public involvement in the design, conduct, or reporting of the trial is not applicable.

Our clusters are townships of Xishui County. The demographic characteristics of Xishui County, including the number and size of townships, are summarized in Table 1. The townships were stratified based on whether their population size exceeded the average size of all townships. Townships within each stratum were randomly allocated in a 1:1 ratio to either the intervention or control arm using a computer-generated randomization sequence. Participant recruitment was based on a comprehensive roster of permanent residents aged 35 years and above, provided by the local government as of May 10, 2024, with individuals selected from each township for enrollment.

**Table 1:** Characteristics of Xishui County, Guizhou

	Xishui County
Total population size (*10,000)	30.20^*^
Number of rural townships	22
Number of villages within rural townships	210
Average rural township size (# of people)	9,518
Number of urban townships	4
Number of communities within urban townships	47
Average urban township size (# of people)	23,150

^*^ Total population size listed in Table 1 was obtained from the Xishui authority. The figures here reflect permanent residents as of May 10th, 2024, and are limited to individuals who had already stayed in Xishui County for three months in 2024 and would continue to stay within the same township for the next year

To identify suspected asthma patients, we used the ECRHS screening questionnaire. The ECRHS screening questionnaire has been translated into Chinese and used in CPHS and regional asthma survey studies [4, 22–24]. The ECRHS screening questionnaire contains items related to asthma symptoms, including whether the individual in question has experienced the following: “wheezed in the past year,” “woken up with tightness in the chest,” “woken by cough,” and “woken by shortness of breath.” The Chinese version of the ECRHS screening questionnaire has a sensitivity of 0.940 and a specificity of 0.710 if the respondent answers “yes” to any question, and it has a sensitivity of 0.860 and a specificity of 0.965 if the respondent answers “yes” to the question asking whether they have “wheezed in the past 12 months.” [2] We categorized participants as suspected asthma patients on the basis of answering “yes” to at least one question on the ECRHS screening questionnaire.

Suspected asthma patients in both arms were notified of their status by a pop-up window upon completing the questionnaire. However, only suspected asthma patients screened within the intervention arm were assigned to an intervention package, while those within the control arm were only invited to complete a face-to-face interview to collect baseline information.

## Methods: participants, interventions, and outcomes

### Setting

Located in the southwestern part of China, Guizhou Province has a smoking prevalence of 37.9% among adults, the highest of all Chinese provinces [25]. Thus, the need for management of tobacco-related NCDs is especially acute in Guizhou Province. This study was implemented across 26 township-level clusters in Xishui County, a mountainous area in northern Guizhou Province. A rural area designated as China’s National Comprehensive Primary Health Experimental Area, Xishui presents a unique combination of policy-driven innovation and socioeconomic challenges. With a GDP per capita 48% below the national average in 2024 and limited human capital development, the county exemplifies resource-constrained rural settings. These conditions, combined with its experimental role in health system transformation, have established Xishui as an ideal setting to evaluate scalable multimorbidity interventions tailored to underserved populations.

### Trial participants (inclusion and exclusion criteria)

All research participants had to be at least 35 years old. Additionally, we required that eligible participants meet the following criteria: 1) answered “yes” to at least one question on the ECRHS screening questionnaire; 2) resided in one township of Xishui County over the prior three months and planned to reside in the same township in the upcoming year; and 3) finished the informed consent.

Individuals with severe cognitive impairments—such as those significantly limiting their ability to comprehend study requirements, make informed decisions, or adhere to intervention protocols—were excluded from enrollment. Similarly, participants with total loss of independence in performing activities of daily living (ADLs) were not eligible. These criteria ensure the study population consists of individuals capable of engaging meaningfully with the intervention and follow-up assessments, while minimizing variables that might skew results. Additional exclusions apply to pregnant individuals and those with medical contraindications to portable spirometry-based lung function testing.

### Intervention

**Fig. 2:**
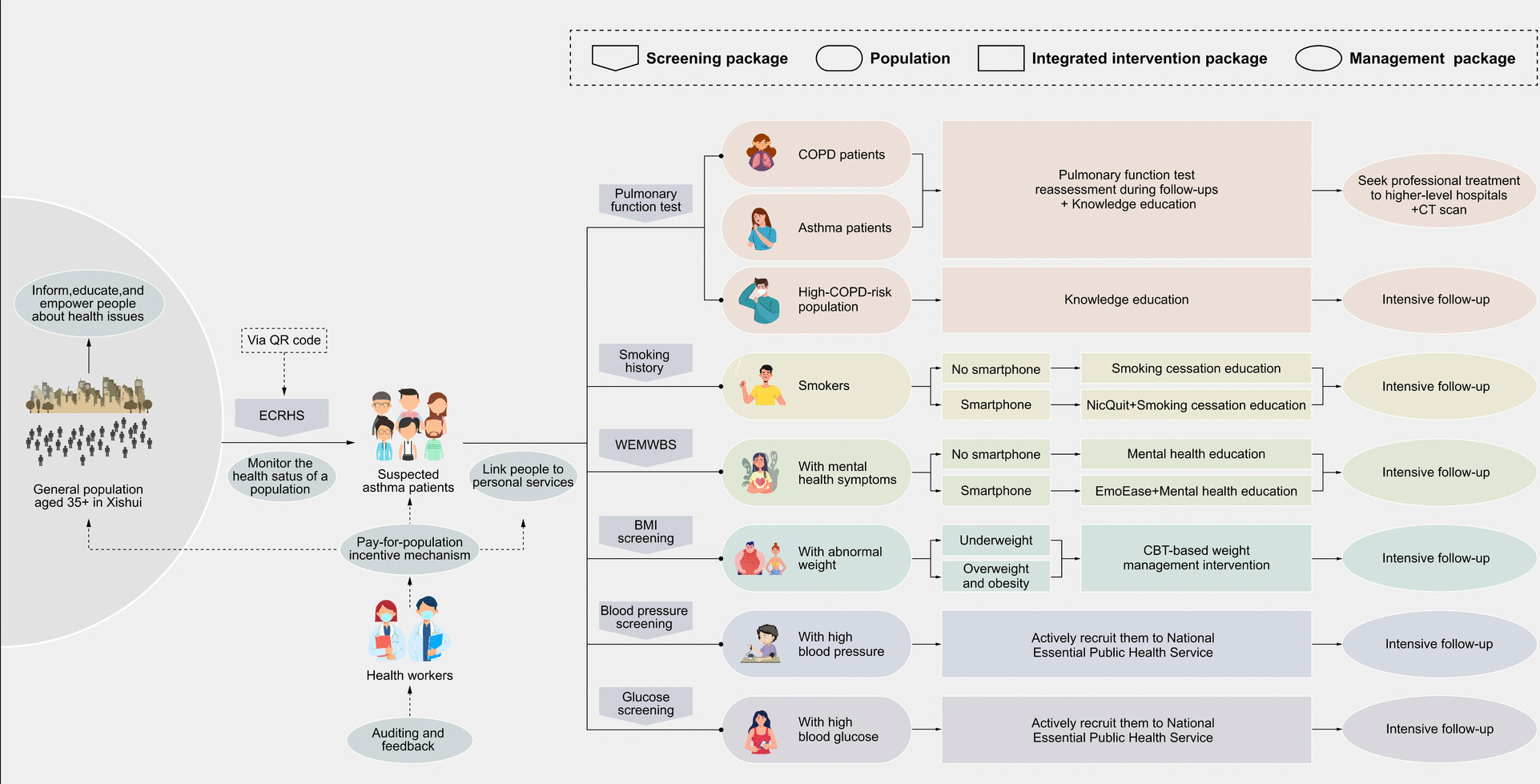
Integrated pathway of the multi-component intervention package. *Note: ECRHS* = *European Community Respiratory Health Survey; COPD* = *chronic obstructive pulmonary disease; WEMWBS* = *Warwick-Edinburgh Mental Well-being Scale; BMI* = *body mass index; CBT* = *cognitive behavioral therapy; CT* = *computed tomography; EmoEase* = *WeChat-based digital mental health intervention based on CBT; NicQuit* = *WeChat-based digital smoking cessation intervention based on CBT*

Individuals randomized to the intervention group gained access to a population medicine multi-component intervention package, developed through an iterative prototyping process and informed by stakeholder consultations. Specific eligibility requirements for each intervention component are detailed in Table 2. Participants assigned to the control group were informed of their "suspected asthma" status and encouraged to complete a face-to-face interview. Post-interview, no active interventions were provided, although individuals in the control group were advised to continue accessing routine medical care. Figure 2 illustrates the structured pathway of the intervention package, including the ECRHS-based screening, stratified risk groups, and corresponding intervention components for suspected asthma patients and co-existing chronic conditions.

**Table 2:** Eligibility criteria for interventions

Population Level	Target Population	Intervention	Eligibility Criteria
General Population	General Population	Health education	Permanent residents
	General Population	Online screening with asthma screening questionnaire	Permanent residents aged 35 years and above
Asthma patients or suspected asthma patients	Suspected asthma patients	Community-based spirometry pulmonary function tests, interpretation of results, and health education	Individuals who answer “yes” to at least one question on the ECRHS screening questionnaire
	Asthma patients or suspected asthma patients who fail to conduct a pulmonary function test	Encouragement to conduct CT scan and seek professional medical treatment	Individuals whose FEV1 improves by ≥ 12% and ≥ 200 mL following bronchodilator administration with 400 μg salbutamol or suspected asthma patients who fail to conduct a pulmonary function test for any reason [25].
	Smokers within suspected asthma patients	Smoking cessation digital health interventions	Currently smoking or have quit within the last 6 months and own a smartphone
	Smokers within suspected asthma patients	Health education to smokers for smoking cessation	Currently smoking or have quit within the last 6 months
	Suspected asthma patients with mental health symptoms	Mental health digital health interventions	Individuals with Warwick Edinburgh Mental Wellbeing Scale (WEMWBS) < 45 who own a smartphone
	Suspected asthma patients with mental health symptoms	Health education	Individuals with WEMWBS < 45
	Suspected asthma patients with hypertension	Hypertension management and education	Three consecutive measurements with average systolic blood pressure ≥ 140 mmHg and/or diastolic blood pressure ≥ 90 mmHg [26].
	Suspected asthma patients with type 2 diabetes mellitus	Diabetes management and education	Fasting blood glucose ≥ 7.0 mmol/L or random blood glucose ≥ 11.1 mmol/L [27].
	Suspected asthma patients with abnormal weight	Weight abnormality interventions	Individuals with BMI < 18.5 kg/m^2^ or BMI ≥ 24.0 kg/m^2^
Health providers	Health providers	Intrinsic incentive mechanism	Primary care providers who engage with the investigation and intervention
	Health providers	Extrinsic incentive mechanism	Primary care providers who engage with the investigation and intervention

All intervention activities were delivered by licensed healthcare professionals at the county, township, and village levels, including general practitioners, village doctors, and public health workers from the Center for Disease Control and Prevention (CDC) of Xishui County. Prior to intervention rollout, all implementers completed a standardized training program organized by the study team, which covered asthma and multimorbidity management, use of spirometry equipment, digital health tools (NicQuit and EmoEase), health education delivery, and participant follow-up procedures. Only healthcare workers who completed all required training sessions and passed the post-training competency assessment were authorized to deliver the intervention. No additional eligibility criteria were applied for participating sites beyond the 26 designated township clusters.

**Fig. 3:**
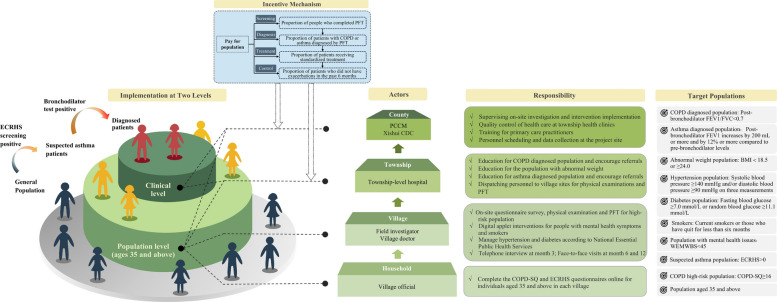
Implementation structure and execution mechanism of the multi-component intervention package. *Note: ECRHS* = *European Community Respiratory Health Survey; COPD* = *chronic obstructive pulmonary disease; FEV1* = *forced expiratory volume in one second; FVC* = *forced vital capacity; BMI* = *body mass index; PFT* = *pulmonary function test; WEMWBS* = *Warwick-Edinburgh Mental Well-being Scale; PCCM* = *Pulmonary and Critical Care Medicine; CDC* = *Center for Disease Control and Prevention*

Figure 3 illustrates the organizational structure, population stratification, incentive design, and responsibility distribution across administrative levels (county, township, village, household) for delivering the intervention in Xishui. This figure also specifies the eligibility criteria for each target subpopulation, clarifying how risk stratification and service delivery were operationalized in the field.

We implemented the following specific interventions as part of the multi-component intervention package:

1. Health education
  Health education is provided to all permanent residents, without any eligibility or risk stratification criteria. The goal is to raise population-wide awareness of asthma and other tobacco-related NCDs, promote early recognition of symptoms, and encourage timely care-seeking behaviors. Educational content covers asthma-related symptoms (such as wheezing and chronic cough), smoking risks, benefits of early spirometry testing, and lifestyle modifications including diet, physical activity, and mental health. Health education is delivered through township-level public campaigns, printed materials (posters, brochures), and short educational videos disseminated via WeChat and village outreach sessions conducted by trained local health workers.
2. Online screening for asthma
  All eligible residents aged 35 years and older are invited to complete an online screening questionnaire via a QR code–linked mobile platform that includes the ECRHS items. Respondents who screen positive on at least one ECRHS item are classified as suspected asthma patients and referred for further evaluation, including spirometry, and tailored interventions. This tool serves as the first triage point for identifying individuals with asthma-related symptoms in the general population and helps guide subsequent intervention pathways.
3. Community-based pulmonary function tests, results interpretation, and health education
  Suspected asthma patients in the intervention arm receive real-time pop-up alerts directing them to a community gathering place for spirometry tests, which are conducted using BH-AX-MAPG spirometry equipment. A spirometry-defined asthma patient refers to an individual whose FEV1 improves by ≥ 12% and ≥ 200 mL following bronchodilator administration with 400 μg salbutamol [26]. Those who screen positive as suspected asthma are referred to the county hospital for computed tomography (CT) and formal diagnosis. In addition, they receive health education on the risks of asthma and how to prevent and manage the disease, delivered through verbal communication by primary healthcare providers and supplemented with printed materials for distribution.
4. Encouragement to seek professional medical treatment in higher-level hospitals for spirometry-defined asthma patients or suspected asthma patients who fail to complete a pulmonary function test
  Participants diagnosed with asthma through spirometry are encouraged to seek professional medical treatment at higher-level hospitals for further diagnosis and management. Some participants fail to complete a pulmonary function test due to contraindications, not understanding the procedure, failing to meet quality control standards, or being unwilling to do the test. Participants who fail to do a pulmonary function test for any reason are encouraged to undergo a CT scan and seek formal diagnosis and medical treatment at a superior hospital.
5. Digital health interventions for smoking cessation
  NicQuit is a WeChat-based digital smoking cessation intervention that includes cognitive-behavioral therapy (CBT) modules focused on smoking cessation strategies, methods for coping with triggers, and reinforcement techniques to maintain abstinence. It is designed for smokers who are currently smoking or have quit within the last six months. The intervention is specifically targeted at individuals familiar with smartphone technology, ensuring accessibility and usability. Personalized notifications and reminders are delivered through the WeChat platform, encouraging participants to regularly engage with the cessation plan and maintain adherence.
6. Health education to smokers for smoking cessation
  Participants in the intervention group receive targeted health education to reinforce the importance of smoking cessation. This education focuses on the health risks associated with smoking and the benefits of quitting, providing participants with evidence-based information and practical advice. This health education is delivered through verbal communication by primary healthcare providers and printed posters or online video messages via WeChat Group for distribution.
7. Digital mental health interventions
  A CBT-based digital mental health intervention, EmoEase [27], is offered to individuals experiencing mental health symptoms (WEMWBS < 45) who have a smartphone. This WeChat-based program includes psychoeducation, mood tracking, guided cognitive-behavioral therapy exercises, and self-regulation techniques. The program emphasizes the link between mental health and respiratory symptoms, aiming to enhance coping, treatment adherence, and sustained behavior change.
8. Health education to suspected asthma patients with mental health issues
  Specialized health education is offered to suspected asthma patients with co-existing mental health symptoms, tailored to their specific challenges. This health education includes guidance on how to manage mental health symptoms and is delivered through verbal communication by trained community health workers or general practitioners and supplemented with printed materials or videos for distribution.
9. Hypertension and diabetes management
  The goal of this intervention is to actively include participating suspected asthma patients whose blood pressure is higher than 140/90 mmHg [28] or whose random blood glucose is higher than 11.1 mmol/L (or fasting blood glucose ≥ 7.0 mmol/L) [29] into the National Essential Public Health Service in China. These participants are also provided health education on hypertension and diabetes through verbal counseling or videos messages by trained community health workers or general practitioners and printed materials for distribution.
10. Weight abnormality interventions
  Individuals with BMI < 18.5 (underweight) or BMI ≥ 24.0 (overweight and obesity) are considered to have weight abnormalities. CBT-based motivational interviewing is used to guide participants in self-identifying weight-related barriers. During the intervention, participants are asked CBT-informed questions designed to make them actively think about the inconveniences and conveniences of being underweight or overweight. Printed materials and video messages are supplemented and distributed.
11. Pay-for-population mechanism
  A novel incentive mechanism combining both extrinsic and intrinsic approaches has been introduced to motivate primary care providers to actively engage in population health interventions. Extrinsic motivation is performance-based and aligns financial rewards with four key stages of care: screening, diagnosis, treatment, and control. At the township level, providers are evaluated based on the proportion of residents aged 35 years and above who complete pulmonary function testing, the proportion of suspected asthma patients identified during initial screening who are subsequently diagnosed with asthma, the proportion of confirmed patients receiving standardized inhaled treatment, and the proportion of patients who have not experienced acute exacerbations in the past six months. The county hospital’s respiratory department and the county CDC are assessed using the same four indicators, with data aggregated across all 13 intervention townships. In addition to offering providers an extrinsic, results-based financial incentive, they are also offered specialized training and capacity-building opportunities in an effort to appeal to their intrinsic motivation, support proactive service delivery, and foster a strong sense of responsibility for population health.

### Outcomes

Primary and secondary endpoints will be assessed at the conclusion of the follow-up period for both trial cohorts. These measurements will focus on individuals randomized to the intervention or control group on the basis of intention-to-treat (ITT). The suspected asthma patient cohort in the intervention arm will undergo longitudinal follow-up, with repeated assessments of endpoints and covariates tracked consistently following their initial ITT assignment. Specifically, key endpoints will be evaluated six months and 12 months after assignment during healthcare provider visits, with the exceptions of disease knowledge, healthcare utilization metrics, and the count of controlled chronic conditions, which will be measured exclusively at the trial’s end (i.e., at 12 months). Table 3 outlines and defines the trial’s primary and secondary endpoints, details the variable specifications for each measure, and describes the methodology used to collect data for every endpoint.

**Table 3:** Primary and secondary outcomes

Primary outcomes	
Number of chronic conditions controlled	Definition: The number of conditions controlled among seven objectively measured chronic health conditions (COPD, asthma, depression symptoms, anxiety symptoms, BMI, hypertension, and type 2 diabetes) Functional form: Counting data Measurement: Through objective physical examination or a validated scale
Lung function testing	Definition: Response to the question “Have you ever had a pulmonary function test?” Functional form: Binary Measurement: Self-reported response
ACT score	Definition: Asthma Control Test (ACT) score Functional form: Continuous Measurement: ACT score, ranging from 0 to 25 with lower scores representing better controlled asthma [30]
Secondary outcomes	
Quality of life indicators	
Self-rated health status	Definition: General self-assessed health status Functional form: Continuous Measurement: EQ-5D scale, ranging from 0 to 1 continuously; 0 represents death, and 1 represents perfect health
Respiratory symptoms	
mMRC score	Definition: Modeified Medical Research Council (mMRC) questionnaire Functional form: Categorical Measurement: Modified Medical Research Council, categorical value, level 0 represents a good respiratory condition, while the maximum level 4 represents a very bad respiratory condition
Asthma care cascade indicators	
Asthma screening	Definition: Response to the question “Have you ever been screened for bronchial asthma by a doctor?” Variable type: Binary Measurement: Self-reported response
Asthma diagnosis	Definition: Response to the question “Have you ever diagnosed as asthma patient by professional physician?” Functional form: Binary Measurement: Self-reported response
Asthma treatment	Definition: Response to the question “Have you followed the treatment therapy from the professional physicians for asthma?” Variable type: Binary Measurement: Self-reported response
Asthma control	Definition: Asthma Control Test (ACT) score ≥ 20, corresponding to “controlled” asthma Variable Type: Binary Measurement: ACT score ranges from 5 to 25, with higher scores representing better asthma control
Knowledge of asthma	
Asthma knowledge	Definition: Responses to excerpted questions of the Patient-completed Asthma Knowledge Questionnaire (PAKQ); selected questions ask about information the general population should know about asthma Functional form: Continuous Measurement: Self-reported responses to questions; scores range from 0 to 7 with higher scores indicating a greater number of correct statements chosen by the respondent
Knowledge and awareness of COPD	
Self-awareness of COPD	Definition: Response to the question “Have you ever been diagnosed with COPD?” Functional form: Binary Measurement: Self-reported response
COPD knowledge	Definition: Responses to excerpted questions of the COPD Knowledge Questionnaire; selected questions ask about information the general population should know about COPD Functional form: Continuous Measurement: Self-reported responses to questions; scores range from 0 to 7 with higher scores indicating a greater number of correct statements chosen by the respondent
Physical health indicators	
FEV_1_ measurement	Definition: Forced Expiratory Volume in one second (L) Functional form: Continuous Measurement: Pulmonary function test, portable spirometry
Blood pressure	Definition: Systolic and diastolic blood pressure (mmHg) Functional form: Continuous Measurement: Omron portable automatic blood pressure monitor
Blood glucose	Definition: Reference standard for average plasma glucose concentration over a period of time, typically reflecting the preceding 8–12 h for fasting blood glucose and the immediate glycemic status for random blood glucose (mmol/L) Functional form: Continuous Measurement: Blood glucose meter
Waist circumference	Definition: Waist circumference (cm) Functional form: Continuous Measurement: Soft measuring tape
BMI	Definition: Body mass index (BMI): weight divided by height squared (kg/m^2^) Functional form: Continuous Measurement: Height and weight were measured using an automated body composition analyzer
Smoking dependence	Definition: A scale that measures the degree of smoking dependence Functional form: Continuous Measurement: Score on the Chinese version of the Fagerström Test for Nicotine Dependence (FTND), which ranges from 0 to 15 with higher scores representing more severe nicotine dependence; additionally measured by score on the Heaviness of Smoking Index (HSI), which ranges from 0 to 6 with higher scores representing worse nicotine dependence
Mental health indicators	
Depression symptoms	Definition: Emotional disorders, including sadness, loss, and anger Functional form: Continuous Measurement: Score on Patient Health Questionnaire-9 items (PHQ-9), which ranges from 0 to 27 with higher scores representing more severe depression symptoms
Anxiety symptoms	Definition: Unpleasant state of inner turmoil Functional form: Continuous Measurement: Score on General Anxiety Disorder-7 (GAD-7), which ranges from 0 to 21 with higher scores representing more severe anxiety symptoms
Warwick-Edinburgh Mental Well-being Scale (WEMWBS)	Definition: Score reflecting overall mental health state Functional form: Continuous Measurement: Score on the Warwick Edinburgh Mental Well-being Scale, which ranges from 14 to 70 with lower scores representing worse general mental health
Care cascade indicators for hypertension and type 2 diabetes mellitus	
High blood pressure (HBP) screening	Definition: Response to the question “Have you ever had your blood pressure measured by a doctor, nurse, or other healthcare professional?” Functional form: Binary Measurement: Self-reported response
HBP diagnosis	Definition: Response to the question “Have you ever been diagnosed with hypertension by a doctor?” Functional form: Binary Measurement: Self-reported response
HBP treatment	Definition: Response to the question “Are you currently taking any antihypertensive medication prescribed by a doctor or other healthcare professional?” Functional form: Binary Measurement: Self-reported response
HBP control	Definition: Blood pressure within the normal range at the end-of-year follow-up Functional form: Binary Measurement: Blood pressure measurement
Type 2 diabetes mellitus (T2DM) screening	Definition: Response to the question “Have you ever had your blood glucose measured by a doctor, nurse, or other healthcare professional?” Functional form: Binary Measurement: Self-reported response
T2DM diagnosis	Definition: Response to the question “Have you ever been diagnosed with T2DM by a doctor?” Functional form: Binary Measurement: Self-reported response
T2DM treatment	Definition: Response to the question “Are you currently receiving a type 2 diabetes treatment plan prescribed by a doctor or other healthcare professional?” Functional form: Binary Measurement: Self-reported response
T2DM control	Definition: Blood glucose within the normal range at the end-of-year follow-up Functional form: Binary Measurement: Blood glucose measurement
Health risk behaviors	
Smoking status	Definition: Response to the question “Do you currently smoke?” Functional form: Binary Measurement: Self-reported response
Amount of smoking	Definition: Average number of cigarettes smoked per day Functional form: Continuous Measurement: Self-reported response
Drinking status	Definition: Frequency of alcohol consumption over the past three months Functional form: Categorical Measurement: Self-reported response; the possible response options are: 0 = Never drink alcohol 1 = Once per month 2 = 2–3 times per month 3 = Once per week 4 = 2–3 times per week 5 = 4–6 times per week 6 = Once per day 7 = Twice per day 8 = More than twice per day 9 = Other, please specify
Sugar consumption	Definition: Frequency of consumption of sugary foods/drinks Functional form: Categorical Measurement: Self-reported response; the possible response options are: 1 = almost every day/daily (6–7 days) 2 = often (4–5 days) 3 = sometimes (2–3 days) 4 = rarely or never (0–1 day)
Salted vegetables consumption	Definition: Frequency of consumption of salted vegetables Functional form: Categorical Measurement: Self-reported response; the possible response options are: 1 = almost every day/daily (6–7 days) 2 = often (4–5 days) 3 = sometimes (2–3 days) 4 = rarely or never (0–1 day)
Vegetable consumption	Definition: Frequency of consumption of vegetables Functional form: Categorical Measurement: Self-reported response; the possible response options are: 1 = almost every day/daily (6–7 days) 2 = often (4–5 days) 3 = sometimes (2–3 days) 4 = rarely or never (0–1 day)
Physical exercise	Definition: Hours of physical exercise per week Functional form: Count Measurement: Self-reported response; the possible response options are: 0 = No related physical activity 1 = Vigorous physical activity (e.g., lifting heavy objects, aerobic exercise, fast cycling) 2 = Moderate physical activity (e.g., lifting light objects, cycling at normal speed, playing doubles tennis; excluding walking) 3 = Light physical activity (e.g., walking)
Healthcare utilization indicators	
Number of outpatient visits	Definition: Number of outpatient visits and type of hospital visited within the past year Functional form: Count Measurement: Survey data
Number of inpatient visits	Definition: Number of inpatient visits and type of hospital visited within the past year Functional form: Count Measurement: Survey data
Medical expenditure within a family over the past year	Definition: Healthcare-related expenditures Functional form: Continuous Measurement: Survey/insurance/outpatient/inpatient data
Socioeconomic profile indicators	
Productivity loss	Definition: Productivity loss due to illness or health problems Functional form: Continuous Measurement: Score on the simplified Chinese version of the Work Productivity and Activity Impairment-General Health (WPAI-GH) (v2.0) questionnaire, with higher scores representing greater impairment and less productivity
Employment status	Definition: Main occupation and type of employment; Functional form: Categorical Measurement: Self-reported response
Family level of annual consumption expenditure	Definition: Annual total household expenditure Functional form: Continuous Measurement: Self-reported responses to a series of questions covering local expenditure categories by the household representative

### Timeline

Our trial commenced on June 17, 2024, with participant recruitment spanning all townships in Xishui County. Individuals in the intervention arm participate in structured evaluations at four distinct time points: at baseline (in-person), at three-month follow-up (telephone), at six-month follow-up (in-person), and at 12-month follow-up (in-person).

At baseline, following informed consent, participants attended a comprehensive in-person assessment. Assessments at baseline and final follow-up include a physical examination and pulmonary function testing, with protocols tailored to specific subgroups: suspected asthma patients undergo pre-bronchodilator spirometry at baseline and 12 months, while confirmed asthma patients receive both pre- and post-bronchodilator spirometry at these time points. Additionally, participants with pre-bronchodilator FEV1/FVC < 70%, indicating airflow limitation, are assessed at baseline, six months, and 12 months using pre-bronchodilator spirometry.

All baseline assessments were conducted under the guidance of trained on-site personnel to ensure precision and adherence to protocol standards. Subsequent follow-ups include a three-month telephone-based check-in to monitor intervention compliance and health conditions and six-month and 12-month in-person visits that repeat key baseline components, including questionnaires, physical exams, and pulmonary function tests.

For the control group, the schedule includes baseline questionnaire administration followed by an on-site survey at the 12-month mark. A visual timeline outlining the prospective cohort visit schedule and data collection points is provided in Table 4.

**Table 4:** Prospective cohort visit and data collection schedule

Timepoint		Trial period				
		Baseline (t_0_)	3 months (t_1_)	6 months (t_2_)	12 months (t_3_)	Close-out (t_4_)^*^
Enrolment						
Sign Informed Consent		X				
QR Code Preliminary Screening		X				
Intervention/comparator						
Multi-component intervention (health education, digital health, spirometry, smoking cessation, etc.)			X	X	X	
Control (routine care only)			X	X	X	
Assessments						
Demographics	Sex, age, ethnicity, education level, marital status, employment status, occupation, income level	X			X	
Risk Factors	Smoking, biomass fuel exposure, occupational exposure, family history (chronic respiratory diseases, including COPD, asthma, and bronchiectasis)	X			X	
Vaccination Inquiry	Influenza vaccination, pneumonia vaccination	X			X	
Disease Information	COPD and asthma diagnosis date	X			X	
	COPD and asthma severity (ventilation function, quality of life score, and ACT score)	X	X	X	X	
	Diabetes and hypertension management	X		X	X	
	Comorbidities	X		X	X	
Treatment	Asthma, COPD, diabetes, and hypertension treatment (care pathway treatment indicators and medication duration)	X		X	X	
	Medication and non-medication adherence (daily use, frequent use, occasional use, use only during exacerbations, or never used)	X	X	X	X	
	Disease-related knowledge				X	
Healthcare Resource Utilization	Out-of-pocket/total costs for asthma, diabetes, and hypertension within one year prior to enrollment or since the last follow-up (medication, outpatient treatment, and hospitalization)				X	
	Healthcare resource utilization within one year prior to enrollment or since the last follow-up (hospital visits and hospitalization days)				X	
	Disease burden of asthma, diabetes, and hypertension within one year prior to enrollment or since the last follow-up				X	
Body Measurements	Height (m), weight (kg), BMI, waist circumference	X		X	X	
Cardiometabolic Indicators	heart rate, blood pressure, blood glucose	X		X	X	
Spirometry	Pre-bronchodilation test	X		X	X	
	Post-bronchodilation test	X			X	
Multi-component Interventions	Implementation of intervention package for eligible participants	X				
	Intervention progress and adherence; re-education of non-adherent participants		X	X	X	
	Evaluation of intervention effectiveness				X	
Intention-to-treat and other analyses						X

^*^The proposed end date of data collection for the last participant is March 31, 2026

### Sample size

The study encompassed all 26 townships within Xishui County, which were randomly assigned to either the intervention or control arm using a 1:1 stratified randomization approach. To ensure representativeness, we recruited approximately 44,000 residents aged 35 years or older across Xishui County. All participants underwent initial screening for suspected asthma via the ECRHS using a QR code-based tool. Accounting for a 10% attrition rate and an estimated 25% positive response rate (based on at least one affirmative ECRHS screening item) among individuals aged 35 years and above, the projected final enrollment target for suspected asthma patients was set at 10,000.

Given the large number of critical secondary endpoints in this study and the lack of robust prior evidence in similar rural and urban community settings, we prioritized the ACT score as one of the three co-primary endpoints to ensure sufficient statistical power and clinical relevance. Since three primary outcomes were specified, we applied Bernoulli adjustment to refine sample size and power estimations. For these calculations, the intraclass correlation coefficient (ICC)—a key parameter for clustered data—was set at 0.0167 (0.05/3), based on findings from the PACK Brazil Study involving asthma patients [31]. This conservative ICC value was adopted to ensure robust sample size estimation.

Sample size determination followed the formula for population-based stratified clustered randomized controlled trials as described by Crespi [30]. We assumed a two-sided significance level of 5% and a target statistical power of 80%. Using these parameters, along with minimum clinically meaningful difference for ACT scores (defined as a three-point mean difference), an estimated uncontrolled asthma prevalence of 35%, and a standard deviation of 5.42, conservative calculations indicated that a minimum of 3,988 suspected asthma patients would need to be enrolled to detect clinically relevant effects.

### Power calculations

Our power analysis demonstrates how minimum detectable differences (MDDs) in ACT scores fluctuate across varying scenarios, assuming a two-tailed significance threshold of 5% and 80% statistical power. We further accounted for potential attrition: 10% of participants may decline trial participation, and an additional 10% may be lost to follow-up for endpoint evaluation. To our knowledge, this represents the first large-scale real-world implementation trial focusing on multimorbidity, with no comparable prior studies available for reference. Consequently, we conducted sensitivity analyses to illustrate how ACT score outcomes might differ across proposed sample sizes. These analyses revealed that our population-based cluster randomized trial design can detect ACT score differences between treatment and control groups ranging from 0.808 to 3.406. Details of power calculation are shown in Table 5. In implementation, stratification was applied before randomization to minimize between-cluster variability.

**Table 5:** Minimum detectable differences in ACT scores

Proposed sample size	ICC = 0.005	ICC = 0.01	ICC = 0.025	ICC = 0.05
2000	1.278	1.655	2.462	3.406
3000	1.043	1.352	2.010	2.781
4000	0.903	1.170	1.741	2.408
5000	0.808	1.047	1.557	2.154

## Methods: assignment of intervention

In this cRCT, participants were assigned at the township level to either the population medicine multi-component intervention or the control arm, with randomization stratified by population size to ensure balanced distribution of community characteristics. A computer-generated randomization sequence was produced by an independent statistician uninvolved in recruitment or intervention delivery, with 13 townships allocated to each arm. Given the intervention’s nature, this is an open-label trial: healthcare providers and research staff are aware of group assignments, while participants were not explicitly informed of their group status or labeled as “intervention recipients” to mitigate expectation bias. The control group receives no additional specified interventions beyond access to routine care throughout the study period.

To strengthen adherence to the intervention protocol, a structured adherence enhancement strategy is embedded across all follow-up stages. Adherence was reinforced through a telephone follow-up at three months and an in-person visit at six months. During these interactions, participants received personalized feedback on their health outcomes—such as spirometry results and ACT scores—accompanied by comparisons to baseline data to highlight progress or areas needing focus. For example, if a participant had not sought professional asthma treatment, root-cause analysis was conducted by revisiting baseline findings and exploring potential barriers (e.g., lack of motivation, challenges accessing healthcare).

Adherence was systematically tracked using validated questionnaires administered at each follow-up, assessing compliance with intervention components (e.g., care-seeking behavior, treatment adherence, app usage frequency, smoking cessation attempts). Automated reminders and tailored counseling were also provided to address gaps in adherence. This iterative process ensures every follow-up serves as an opportunity to motivate participants, troubleshoot challenges, and adapt strategies to sustain engagement over the trial’s duration.

## Methods: data collection, management, and analysis

### Data collection plan

To comprehensively evaluate the impact of the multi-component intervention, trial data are being collected across multiple domains (Table 4). During the baseline phase, all participants completed a detailed, structured questionnaire covering demographics and socioeconomic status, quality of life, lifestyle and risk behaviors, medical history, mental health status (via PHQ-9 for depression, GAD-7 for anxiety, and the Warwick-Edinburgh Mental Well-being Scale [WEMWBS]), and productivity loss. In addition to providing self-reported data, participants underwent physical assessments, including measurements of height, weight, BMI, waist circumference, heart rate, blood pressure, and blood glucose levels. Respiratory health was evaluated through spirometry to assess lung function and detect airflow limitations.

For all individuals suspected of having asthma, pre-bronchodilator spirometry was performed at baseline and again performed at 12 months to monitor longitudinal changes in lung function. Among participants confirmed to have asthma at baseline, both pre- and post-bronchodilator spirometry were conducted at baseline and again at six months (pre-bronchodilator only) and 12 months.

Follow-up data collection occurs at three time points: three months (telephone-based), six months (in-person), and 12 months (in-person). At each follow-up visit, participants repeat key components of the baseline assessment, including self-reported data on asthma symptoms and management (e.g., ACT, diagnosis status, treatment regimens, and asthma control levels), high-risk behaviors (e.g., daily cigarette consumption, smoking dependence, and alcohol intake), and chronic disease status (blood pressure, blood glucose, and BMI). Mental health is re-evaluated using the same validated scales (PHQ-9, GAD-7, and WEMWBS), and health-related quality of life is measured via the EQ-5D-5L scale. Lifestyle behaviors—such as physical activity levels, alcohol consumption, and dietary patterns (including sugar, salted vegetable, and fresh vegetable intake)—are monitored through self-reports. Additionally, data on healthcare resource utilization (e.g., outpatient and inpatient visits) are recorded.

All data collection is conducted by trained field staff following standardized protocols to ensure accuracy and reliability. Collected data are entered electronically into a secure electronic data capture (EDC) system equipped with built-in validation checks to minimize entry errors.

### Data management

This investigation utilizes an EDC platform to enable immediate data capture, automated checks, and secure storage. Information is gathered by trained research personnel using tablet-enabled digital questionnaires, which automatically sync with a central repository. The EDC system is equipped with range validations, logical coherence checks, and missing data notifications to reduce errors; any highlighted discrepancies are swiftly evaluated by the data management team. Data collection instruments follow uniform, pre-coded formats to streamline demographic, clinical, and behavioral assessments, while biophysical measurements (e.g., lung function, blood pressure, BMI, and blood glucose levels) are input directly into the system. Spirometry results are sent from handheld devices and undergo automated quality assurance processes to confirm validity.

To safeguard participant confidentiality, all data are anonymized using numeric coding and stored in a password-secured database exclusively reserved for analytical purposes by the research team. Access to this repository is restricted to a small group of authorized individuals, including the principal investigator (PI), co-investigators, biostatisticians, and data analysts.

### Statistical analysis

Statistical analyses will be conducted within an intention-to-treat (ITT) framework, whereby all participants are evaluated with respect to outcome measures according to their original random assignment. The primary analytical approach will utilize a generalized linear mixed model (GLMM), supplemented by a sensitivity analysis to account for potential missing data issues. To enhance statistical precision, we will compute both unadjusted and covariate-adjusted effect estimates, integrating baseline covariates including age, sex, smoking history, comorbid conditions, socioeconomic status, and baseline measurements of the outcome variables. Additionally, standard errors will be clustered at the township level to correct for potential within-group correlation, thereby improving the robustness of inference. Adjustment for multiple testing will be applied using the Bonferroni correction for primary outcomes and the Benjamini–Hochberg procedure for secondary outcomes. For Benjamini–Hochberg procedure for secondary outcomes, we set our false discovery rate as 0.05 and plan to rank the *p*-values of all secondary outcomes listed from the smallest to the largest.

## Methods: monitoring

To safeguard scientific integrity and ensure independent, safety-focused oversight, we established an independent Data and Safety Monitoring Board (DSMB). The DSMB comprises leading experts in public health, epidemiology, statistics, clinical research, and respiratory disease. The DSMB convened three meetings in which baseline and follow-up descriptive analyses for the intervention arm were evaluated for process monitoring. The last DSMB meeting was in February 2026, in which the statistician presented a formal interim analysis based on temporary data of the 12-month follow-up for both the intervention and the control arms.

During these meetings, the research team provided updates on trial execution, including reviews of how well the intervention components were being followed, logistical hurdles encountered, and assessments of data completeness and quality. In February 2026, our biostatisticians also shared emerging effect estimates from both the intervention and control groups with the DSMB. The DSMB then evaluated whether the results fell into one of four scenarios:

1. Null findings, where no detectable effect of the intervention was observed, potentially prompting discussion about study termination;
2. Statistically significant results, indicating the primary objectives had been met, which could lead to study termination;
3. Emerging but statistically insignificant effects, suggesting the trial may require further observations if applicable. However, the study will be terminated if field workers confirm all eligible participants have been contacted and no additional participants remain to undergo follow-up assessments;
4. If deaths or severe adverse effects were reported, these would be immediately conveyed to the DSMB. The DSMB would determine if the deaths or adverse effects were related to the intervention and advise if the study should be stopped immediately.

## Ethics and dissemination

### Research ethics approval

This study received ethical approval from the Peking Union Medical College Ethics Committee. To protect participant privacy, all identifying information has been removed from the dataset, and data are anonymized before analysis. Participants have the right to withdraw from the study at any point, with no consequences for their standard healthcare access. The study is committed to upholding the highest standards of ethical research conduct, ensuring that all participants are treated respectfully and that their health and personal information are safeguarded throughout the research process. Ethics approval has been obtained from the Ethics Commission of the Medical Faculty at Peking Union Medical College (approval number: CAMS&PUMC-IEC-2024–041), and informed consent was collected from all participants. A continuing ethics review was completed in June 2025, and updated approval was granted under approval number CAMS&PUMC-IEC-2025–062.

### Plans for communicating important protocol amendments to relevant parties

The Ethics Commission of the Medical Faculty at Peking Union Medical College was consulted and informed of all protocol changes requiring its review and approval. After securing Ethics Committee clearance but prior to implementation, all research staff were briefed on the revised protocol details.

Within this trial, a necessary adjustment was made to the secondary outcome measures. While modifications to outcomes are typically not recommended, this change was driven by an unavoidable regulatory constraint. Originally, we intended to assess healthcare utilization—including outpatient visits, inpatient admissions, and medical expenditures—using electronic medical records from healthcare facilities in Xishui County. However, unforeseen government regulations prevented us from reaching agreement to access these data. This revision was formally approved by the Ethics Commission, and all research personnel were notified prior to implementation to maintain uniformity in data handling and analysis.

### Consent and withdrawal

We provided written study information and the informed consent form to eligible individuals. The field workers explained the study’s aims and detailed procedures—in the presence of a witness if required. We provided sufficient time for our participants to decide whether or not to participate in the study. Participants were given the opportunity to inquire about details of the study, and responses were provided. For illiterate participants, we obtained a thumbprint signing and a witness’s signature to document consent before enrolment.

We informed eligible participants about the risks and benefits of participation in the trial. A decision not to participate in the study will not bear any further consequences for the individual. Every participant is free to refuse or discontinue data collection at any stage. Importantly, participation does not require relinquishing any concomitant care. While there are no special criteria for modifying or discontinuing allocated interventions, we will document and report reasons for attrition in future publications.

### Confidentiality

Throughout the study, all data have been handled with complete confidentiality, and data collection has conformed to requirements of the national legislations on data protection. We are storing digital data on password-protected files in the Center of the Chinese Academy of Medical Sciences. Data are available exclusively to the research team members for complete confidentiality. Third parties may only receive anonymized data only for research purposes. Informed consent forms, laboratory books, and other participant-related documents are safely being stored during study’s conduct and will be stored subsequently at the Co-Principal Investigator’s office premises.

### Dissemination

Upon completion of the trial, participant-related information and results will be provided to the participants themselves or to their families. The trial has gained public attention since it was first introduced in the *Lancet* Commission on Investing in Health. We will disseminate the results of the trial in international peer-review journals and at other renowned international academic conferences.

## Discussion

The POPMIX-Asthma trial is based on population medicine principles [1]. Unlike traditional clinical trials that focus on individual patients, it aims to improve the overall health status of an entire target population. It does so by focusing on suspected asthma patients rather than diagnosed patients and on multimorbidity rather than asthma as a standalone disease. By targeting suspected asthma patients, we aim to address unmet healthcare need and identify more undiagnosed individuals in a low-resource setting. This exemplifies a shift among medical professionals from patient-centered care to population-centered care, expanding their scope of responsibility beyond the clinic to proactively address unmet healthcare need in the community.

By focusing on multimorbidity rather than solely asthma, we encourage medical workers to care for all common diseases at the population level, as multimorbidity status is common among adults and particularly the elderly. We selected “number of chronic diseases controlled” as a primary outcome to demonstrate that our trial is concerned with not only asthma but also common co-occurring conditions, such as hypertension, diabetes, smoking dependence, abnormal BMI, COPD, depression and anxiety symptoms. We selected “ACT score” as a primary outcome to enable evaluation of whether a population-based integrated intervention package targeting suspected asthma patients generates positive effects on asthma control. Finally, we selected whether participants received a lung function test as a primary outcome in accordance with our trial’s interest in implementation science and to demonstrate one of the trial’s key goals, i.e., facilitating early diagnosis and appropriate management of suspected asthma patients.

To strengthen the motivation of health workers supporting population health, we designed a pay-for-population incentive mechanism to provide extrinsic motivation for improving the care cascades for asthma and other tobacco-related NCDs. The trial also appeals to intrinsic motivation via intensive professional trainings. By combining intrinsic and extrinsic motivations for health workers, we seek to transition from theory to action in emphasizing the responsibility of health professionals to care for population health as well as individual health.

The objective of this trial is to improve the health of suspected asthma patients, which was defined by ECRHS screening questionnaire and reported symptoms related to asthma, like wheezing. Although asthma is a complex disease where medical professionals at low-resource settings are usually difficult to diagnose [4–6], we aim to encourage suspected asthma patients who have reported related symptoms to conduct formal diagnosis and seek treatment at hospitals. Thus, we prefer to define our trial as an implementation science trial to bring suspected patients on the right track of formal diagnosis, treatment, and management.

We included many secondary outcomes in the trial to evaluate the effectiveness of our integrated management intervention package on common co-occurring conditions, including COPD, tobacco dependence, abnormal weight, hypertension, type 2 diabetes, and mental health symptoms. To design the integrated management intervention, we follow the modular approach introduced by The *Lancet *Commission on Investing in Health, in which all of the integrated intervention strategies were proven cost-effective [2]. Our trial has been listed as an important case study in the Commission Report.

Given the complexity and overlapping manifestations of asthma as well as the limited healthcare capabilities typical of low-resource settings, we only conducted lung function testing on suspected asthma patients and not bronchial provocative testing (BPT). BPT is generally not suitable, feasible, or affordable in many resource-limited township hospitals. Our working definition of asthma within this trial can only capture asthma with airflow limitation. This is an important subset of asthma patients, especially in China where 26.2% of adults with asthma had airflow limitation [2, 10]. This figure is similar to the estimate of 21.7% of adults with asthma reported from six low- and middle-income countries [4, 10]. The relatively high proportion of asthma patients experiencing airflow limitation might show an underuse of inhaled corticosteroids [2].

The findings from our trial highlight the need for a paradigm shift in the role of primary and community health workers within population health management. In conventional health systems, frontline providers often operate passively, waiting for patients to present at clinics. This trial introduces mechanisms such as pay-for-population, performance-linked process metrics, and home-based outreach to actively engage providers in population screening, health promotion, and continuous care. This transformation—from a patient-centered, reactive model to a proactive, population-centered approach—is at the heart of population medicine. We believe this shift has the potential to reshape public health governance not only in China but also in other low- and middle-income countries striving for equitable, sustainable health system reform.

## Conclusion

In conclusion, the POPMIX-Asthma trial represents a pioneering effort to integrate population medicine principles into multimorbidity management for suspected asthma patients in resource-limited settings. By combining community-based screening, digital health interventions, team-based care, and innovative incentive mechanisms, this trial aims to improve early detection, facilitate timely diagnosis, and enhance comprehensive disease management. The outcomes of this study are expected to provide robust evidence for scaling up integrated population-based interventions, inform national and international health policies, and contribute to closing gaps in chronic disease care. Ultimately, the trial underscores the potential of population medicine to transform healthcare delivery and promote equitable and sustainable improvements in population health.

## Supplementary Information

Supplementary Material 1.

## Data Availability

The datasets used and/or analysed in this study are available from the PI on reasonable request.

## Abbreviations

ACT: Asthma Control Test
ADL: Activities of daily living
BMI: Body Mass Index
BPT: Bronchial Provocative Testing
CAT: COPD Assessment Test
CBT: Cognitive behavioral therapy
CDC: Center for Disease Control and Prevention
COPD: Chronic obstructive pulmonary disease
CPHS: China Pulmonary Health Survey
cRCT: Cluster-randomized controlled trial
CT: Computed tomography
DALY: Disability-adjusted life years
DSMB: Data and safety monitoring board
ECRHS: European Community Respiratory Health Survey
EDC: Electronic data capture
EISL: International Study of Wheezing in Infants
EQ-5D-5L: EuroQol 5-Dimension 5-Level
FEV1: Forced expiratory volume in 1 s
FTND: Fagerström Test for Nicotine Dependence
FVC: Forced vital capacity
GAD-7: General Anxiety Disorder-7
GLMM: Generalized linear mixed model
HBP: High blood pressure
HSI: Heaviness of Smoking Index
ICC: Intraclass correlation coefficient
ISAAC: International Study of Asthma and Allergies in Childhood
ITT: Intention-to-treat
MDD: Minimum detectable difference
mMRC: Modified Medical Research Council
NCD: Non-communicable disease
PAKQ: Patient-completed Asthma Knowledge Questionnaire
PCCM: Pulmonary and Critical Care Medicine
PFT: Pulmonary function test
PHQ-9: Patient Health Questionnaire-9 items
PI: Principal investigator
POPMIX: Population Medicine Multimorbidity Interventions in Xishui
SGRQ: Saint George Respiratory Questionnaire
SPIRIT: Standard Protocol Items: Recommendations for Interventional Trials
T2DM: Type 2 diabetes mellitus
WEMWBS: Warwick-Edinburgh Mental Well-being Scale
WHS: World Health Survey
WPAI-GH: Work Productivity and Activity Impairment-General Health
